# Musculoskeletal symptoms among teleworkers assessed using validated tools: a systematic review and single-arm meta-analysis

**DOI:** 10.3389/fpubh.2026.1941061

**Published:** 2026-09-28

**Authors:** Monika Zawadka-Kunikowska, Mateusz Tomanek, Anna Skarpańska-Stejnborn, Zofia Adamczyk, Wojciech Kaźmierczak, Łukasz Rzepiński, Stanisław Krajewski, Mirosława Cieślicka

**Affiliations:** 1 Department of Human Physiology, Nicolaus Copernicus University Ludwik Rydygier Collegium Medicum in Bydgoszcz, Bydgoszcz, Poland; 2 Department of Business Excellence Institute of Management and Quality Sciences Faculty of Economic Sciences and Management, Nicolaus Copernicus University, Toruń, Poland; 3 Department of Biological Sciences, Faculty of Sport Sciences in Gorzow Wielkopolski, Poznan University of Physical Education, Gorzów Wielkopolski, Poland; 4 SRC Audiology and Foniatry, Nicolaus Copernicus University Ludwik Rydygier Collegium Medicum in Bydgoszcz, Bydgoszcz, Poland; 5 Department of Clinical Medicine, Faculty of Medicine, University of Science and Technology, Bydgoszcz, Poland; 6 Department of Neurology, 10th Military Research Hospital and Polyclinic, Bydgoszcz, Poland; 7 Department of Neurosurgery, 10th Military Research Hospital and Polyclinic, Bydgoszcz, Poland

**Keywords:** ergonomic risk, meta-analysis, musculoskeletal symptoms, Nordic musculoskeletal questionnaire, Rapid Office Strain Assessment (ROSA), remote work, telework

## Abstract

**Background:**

Work-related musculoskeletal disorders are common in teleworkers, but reported prevalence rates are inconsistent. This study aimed to estimate the pooled prevalence of musculoskeletal symptoms and to quantify ergonomic risk in teleworkers through single-arm meta-analysis.

**Methods:**

Two reviewers searched PubMed, Scopus, Web of Science, and EBSCO from database inception to March 19, 2026 for observational studies reporting musculoskeletal symptoms or ergonomic risk in teleworkers assessed using validated instruments. A random-effects single-arm meta-analysis was performed to calculate pooled prevalence estimates and pooled mean scores with 95% confidence intervals (CI) and prediction intervals (PI).

**Results:**

Twenty-one studies were included. Nordic-based questionnaires (*n* = 13) were the most frequently used instruments for assessing musculoskeletal symptoms, while the Rapid Office Strain Assessment (ROSA; *n* = 6) and Maastricht Upper Extremity Questionnaire (*n* = 4) were the most commonly used ergonomic assessment tools. Most studies were rated as moderate quality based on the Newcastle–Ottawa Scale. Pooled prevalence analysis (5 studies, *n* = 2,215 teleworkers) showed the highest prevalence for neck symptoms (54.9%; 95%CI 20.8–85.0%), followed by shoulder symptoms (52.8%; 95%CI 34.1–70.7%) and low back symptoms (49.0%; 95%CI 25.4–73.1%), with substantial between-study heterogeneity. Pooled ROSA total scores (6 studies, n = 1,086 teleworkers) indicated moderate-to-high ergonomic risk (mean score, 4.27; PI 2.58–5.95). Regarding ROSA components (5 studies, A–C), section A (chair) yielded the highest mean score (4.07; 95% CI 2.98–5.16), followed by section C (monitor and telephone: 3.04; 95% CI 2.69–3.40) and section B (keyboard and mouse: 2.18; 95% CI 1.80–2.56).

**Conclusion:**

Very low-certainty evidence suggests that neck, shoulder, and low back symptoms are prevalent among teleworkers. Pooled ROSA estimates indicated moderate ergonomic risk, supporting the need for preventive ergonomic strategies and standardized assessment methods in teleworking populations.

**Systematic review registration:**

https://doi.org/10.17605/OSF.IO/EMVYG

## 1 Introduction

An estimated 1.71 billion people are affected by musculoskeletal disorders (MSDs) worldwide, making them one of the leading causes of disability (1). Work-related musculoskeletal disorders (WMSDs) are the most frequently reported occupational health problems in the European Union, accounting for about two-fifths of all occupational diseases in EU Member States (2). WMSDs affect approximately 70–80% of office workers and contribute substantially to disability, sickness absence, and reduced work capacity (3, 4). Across populations, women consistently exhibit higher prevalence rates of MSDs than men, and the burden of MSDs increases with age. Beyond their health impact, these disorders impose considerable economic and social costs on individuals, employers, and healthcare systems (1, 2, 5).

MSDs are multifactorial conditions arising from complex interactions between physical, psychosocial, organizational, and individual determinants (6). Physical and ergonomic factors are known to be important contributors to the development of WMSDs. In this context, changes in work organization and the growing use of digital technologies, particularly the shift towards telework, have emerged as important determinants of MSD risk (7).

The COVID-19 pandemic triggered a rapid expansion of telework, which has since become an established component of modern employment (7). Remote work in Europe increased from approximately 14% before the COVID-19 pandemic to around 22% in 2023, with hybrid arrangements now representing the dominant form of remote work (7–9). Preferences for regular telework also continue to increase, with approximately half of both men and women expressing a desire to work from home several times a week (9).

While telework may provide greater autonomy and flexibility, it is also associated with prolonged sitting, extended working hours, and time pressure, all of which may adversely affect workers’ health and wellbeing (7, 10). These factors may contribute to the development or exacerbation of MSDs, particularly in the neck, upper limbs, and back. With the continued expansion of remote and hybrid work, telework-related MSDs are expected to become an increasing occupational and public health concern, highlighting the need for effective preventive and ergonomic strategies (11).

Telework may also increase ergonomic risks, as working outside the employer’s premises complicates ergonomic assessment and the implementation of occupational safety and health standards (7). However, the results of research on ergonomic risk in desk-based teleworkers are inconsistent. While some studies report low risk, moderate-to-high ergonomic risk predominates across most occupational groups (12–17). Workstation characteristics, intensive computer use, psychosocial demands, and break patterns have been shown to be associated with musculoskeletal symptoms in teleworkers (18–21), most commonly low back and neck pain (18, 19). However, interpretation of existing data is complicated by the use of diverse validated instruments and outcome measures, and existing reviews have primarily focused on describing symptom characteristics and risk factors rather than performing formal meta-analyses of musculoskeletal symptom prevalence and ergonomic risk (18, 19) Therefore, here we aimed to comprehensively evaluate and synthesize the available evidence on musculoskeletal symptoms and ergonomic risk among teleworkers. Our specific objective was to quantify musculoskeletal symptom prevalence and ergonomic risk measured using validated assessment tools through a single-arm meta-analysis.

## 2 Methods

This systematic review and meta-analysis was conducted in accordance with the updated PRISMA 2020 (Preferred Reporting Items for Systematic Reviews and Meta-Analyses) guidelines (22) and Meta-analysis of Observational Studies in Epidemiology (MOOSE) guidelines (23). The protocol was retrospectively registered in the Open Science Framework^1^. No methodological changes were made following registration.

### 2.1 Search strategy

Two independent reviewers (MZK and MT) searched PubMed, Web of Science, Scopus, and EBSCO (MEDLINE Ultimate, Health Source: Nursing) from database inception until May 29, 2025, with an updated search conducted on March 19, 2026, to identify newly published studies.

The PICO framework was defined as follows: P (participants): adults (≥18 years) performing telework (remote or home-based work); I (intervention): not applicable; C (comparator): not applicable (single-arm synthesis); O (outcomes): prevalence and continuous measures (mean values and corresponding standard deviations) of musculoskeletal symptoms and ergonomic risk assessed using validated instruments; S (study design): observational studies (cross-sectional, cohort, and case–control studies). The detailed search strategy and search terms for each database are presented in the Supplementary materials.

### 2.2 Eligibility criteria

Studies were included if they met the following criteria: (i) adults (≥18 years) performing telework (remote or home-based work) as workers/employees, defined as predominantly sedentary, desk-based tasks using information and communication technologies (ICT), including administrative or teaching activities during the COVID-19 pandemic; because definitions of telework varied across studies, all forms of predominantly home-based or remote ICT-supported work were considered eligible; (ii) assessment of musculoskeletal outcomes using validated or standardized instruments (e.g., Nordic Musculoskeletal Questionnaire [NMQ], Cornell Musculoskeletal Discomfort Questionnaire [CMDQ], Rapid Office Strain Assessment [ROSA], Maastricht Upper Extremity Questionnaire [MUEQ]), with outcomes reported as prevalence, frequency, intensity, or mean (SD) values; and (iii) observational study designs (e.g., cross-sectional, cohort, or pre–post studies) published as full-text articles in English or Polish.

Studies were excluded if they: (i) were reviews, letters, conference abstracts, or posters; (ii) were not available as full-text articles in English or Polish; (iii) reported only subjective musculoskeletal complaints without the use of structured or validated assessment tools; or (iv) were conducted in student populations.

### 2.3 Study selection and data extraction

Study selection involved initial screening of titles and abstracts, followed by full-text assessment of potentially eligible studies. Relevant data were subsequently extracted from the included full-text articles. Data were extracted using a standardized extraction form developed for this review. Two authors (MZK and MT) independently extracted data, including the first author’s name, year of publication, country of origin, study design (cross-sectional or prospective), population characteristics, sample size, percentage of female participants, participant age [mean (SD) or range], and body mass index (BMI).

Data on telework exposure were also extracted, including definition of telework, telework period, and exposure characteristics (e.g., intensity thresholds, duration criteria, or proportion of telework). Additionally, information on workload (e.g., working hours, proportion of telework, or sitting/computer time), length of service, and follow-up duration (where applicable) was collected.

Outcome-related data included tools used to assess musculoskeletal symptoms (e.g., NMQ, CMDQ, and region-specific scales) and ergonomic risk (e.g., ROSA, MUEQ). Mean values and SDs were extracted for continuous outcomes, as well as event counts and total sample sizes for prevalence outcomes. Disagreements between reviewers were resolved through discussion with a third reviewer (MC).

### 2.4 Methodological quality, risk of bias

Methodological quality and risk of bias were assessed using the Joanna Briggs Institute (JBI) critical appraisal tools (24) and the Newcastle–Ottawa Scale (NOS) (25). For JBI, scores of 7–8/8 (cross-sectional) and 10–11/11 (cohort) indicated low risk of bias, 5–6/8 and 7–9/11 moderate risk, and ≤4/8 and ≤6/11 high risk, respectively (24). For NOS, scores of 7–9/9 indicated high quality, 4–6/9 moderate quality, and 1–3/9 low quality (25). Assessments were independently performed by two reviewers (MZK and MT) and were verified by a third reviewer (MC).

### 2.5 Outcomes

The primary outcomes of interest were ergonomic risk scores [mean (SD)] and event rates for MSK symptoms assessed using validated instruments. The following validated instruments were identified across the included studies: NMQ, CMDQ, ROSA, Rapid Upper Limb Assessment (RULA), Rapid Entire Body Assessment (REBA), MUEQ, Roland–Morris Disability Questionnaire (RMDQ), Lower Extremity Functional Scale (LEFS), Quick Disabilities of the Arm, Shoulder and Hand questionnaire (QuickDASH), Neck Disability Index (NDI), Oswestry Low Back Disability Questionnaire (ODI), Visual Analogue Scale (VAS), Numeric Rating Scale (NRS), and other standardized ergonomic and pain-related assessment tools.

### 2.6 Data synthesis

All analyses were performed using Comprehensive Meta-Analysis software v4.0. A single-arm meta-analytic approach was used to pool prevalence estimates and continuous ergonomic risk outcomes across teleworking populations. Outcomes reported in at least three studies using comparable validated instruments and outcome definitions were included in the meta-analysis, whereas outcomes reported in fewer than three studies were synthesized narratively. Pooled estimates were calculated using a random-effects model (DerSimonian–Laird estimator for random effects) (26, 27). For prevalence outcomes, pooled event rates were calculated using logit-transformed proportions and corresponding standard errors. Continuous outcomes were pooled using mean values, corresponding SDs, and sample sizes. All pooled estimates were presented with 95% confidence intervals (95% CIs). Heterogeneity across studies was assessed using the *Q* statistic and the *I*^2^ statistic, with a *p*-value < 0.05 considered indicative of significant heterogeneity. *I*^2^ thresholds were defined as: ≥75% (high), 50–74% (moderate), 25–49% (low), and <25% (none). Prediction intervals (PI) were additionally calculated to estimate the expected range of true effects across comparable populations under the random-effects model. The PI estimates the range of true effects expected in comparable studies and provides information on the extent of between-study heterogeneity (28). Sensitivity analysis was conducted to explore sources of heterogeneity when I^2^ exceeded 50% (29). Publication bias was not formally assessed, as fewer than 10 studies were included in each meta-analysis. Statistical significance was set at a *p*-value < 0.05. We used the Grading of Recommendations Assessment, Development and Evaluation (GRADE) approach to assess the certainty of evidence for each outcome included in the meta-analysis GRADE classifies the certainty of evidence as high, moderate, low, or very low based on risk of bias, inconsistency, indirectness, imprecision, and publication bias. The certainty of evidence was initially rated as high and downgraded when concerns were identified in any GRADE domain (30).

## 3 Results

Of the 247 studies initially identified, 168 records were screened and 26 full texts were assessed for eligibility. After exclusion of five studies reporting the wrong outcome, 21 studies were included in the review. The study selection process is presented in the PRISMA flow chart (Figure 1). Among the 21 included studies, 19 were cross-sectional and 2 were prospective.

**Figure 1:**
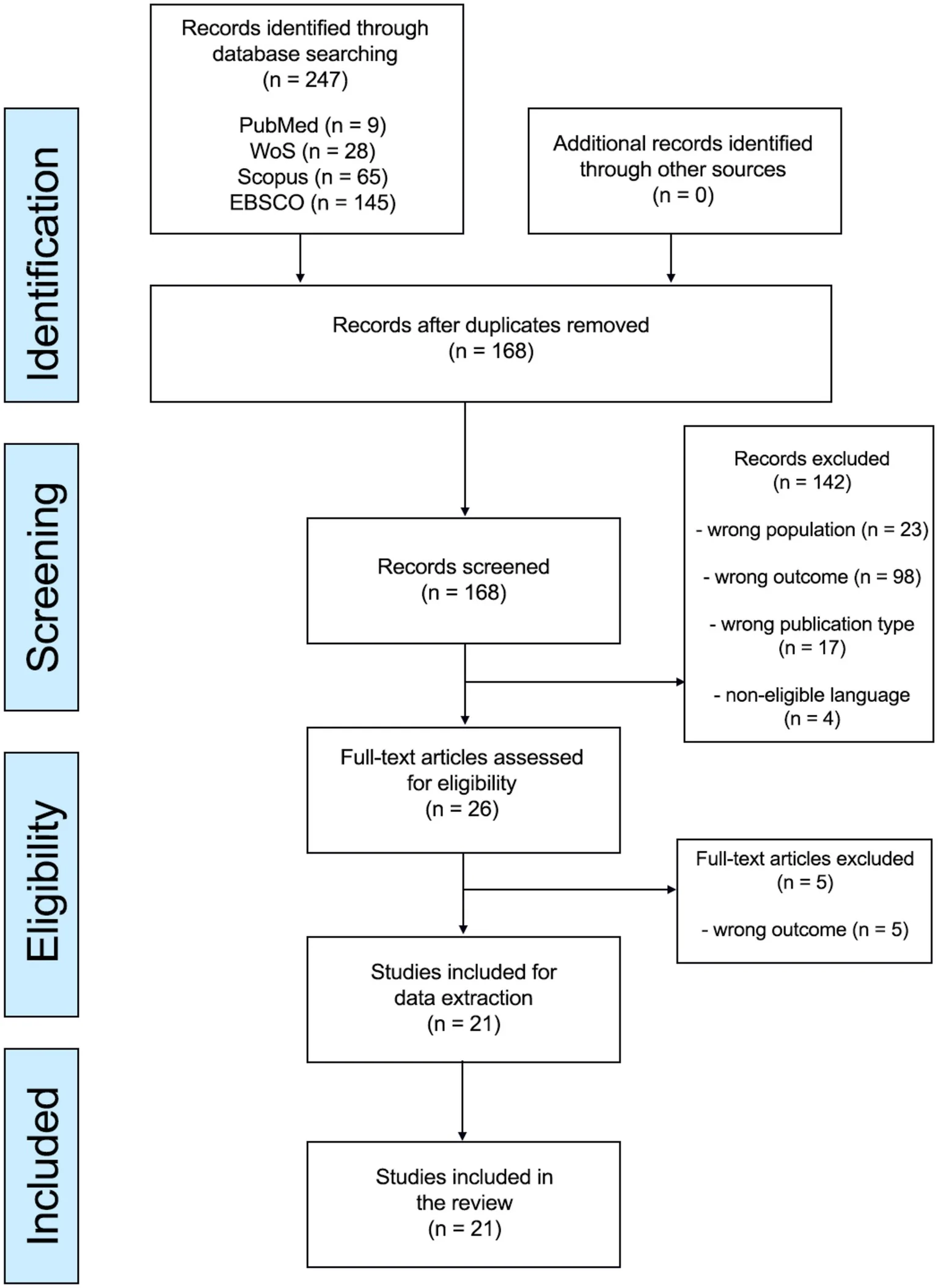
PRISMA flow chart.

### 3.1 Descriptive characteristics

Tables 1, 2 show the main characteristics of the included studies, which were published between 2020 and 2026. None were conducted exclusively before the COVID-19 pandemic. Most studies (*n* = 18) collected data during or after the onset of the pandemic (12–16, 20, 21, 31–41), while three did not report the timing of data collection (17, 42, 43).

**Table 1:** Descriptive characteristics of the included studies.

Study	Country	Design	Telework period	Telework exposure (definition, intensity, and inclusion criteria)	Population	Total sample, n	Mean age, years (SD)	Total % Female	BMI (kg/m^2^)	Mean workload, hours/day or hours/week (SD)	Mean length of service, years (SD)
Soto-Martínez 2025 (12)	Chile	Cross-sectional	2021	Unrestricted telework	Teachers	51	40.0 (7.8)	58.8	26.6	NR	5.3 (3.9)
Ambrosio 2023 (31)	Italy	Cross-sectional	2021–2022	Remote work (home-based); last 18 months	Mixed	93	47.4 (10.1)	47.3	NR	38.09 (13.3) h/week	NR
Takasaki 2024 (36)	Japan	Cross-sectional	2023	Home-based telework: 100, >70%, 50–70%, 30–50%, <30%; 100% or >70% was classified as full/almost full telework.	Desk workers	2700^a-d^	49.1 (8.4)ᵃ; 43.5 (9.5)ᵇ; 48.8 (8.7)ᶜ; 42.8 (9.6)ᵈ	50	23.6 (3.7)^a^ 21.1 (3.6)^b^ 23.8 (4.1)^c^ 20.6 (3.2)^d^	NR	NR
Bilecik 2023 (41)	Turkey	Prospective cohort	2020–2021	Transition to WFH (shift from on-site to home work)	Teachers	679	41.7 (9.6)	67.9	NR	21.2 (9.2) h/week (baseline)vs. 21.6 (9.5) h/week (1 year)	17.0 (7.7)
Houle 2021 (34)	Canada	Prospective cohort	2020	Telework via ICT; ≥1 week full-time telework	Office workers	162 Responders: 109 Non-responders: 53	36.2 (11.1)^e^	63.3^e^	24.9^e^	5.4 h/day^e^	NR
Rodríguez-Nogueira 2020 (35)	Spain	Cross-sectional	2020	WFH during COVID-19	University staff	472	46.4 ^f^	60	NR	Sitting time: 6.9 h/day^f^	NR
Filho 2023 (15)	Brazil	Cross-sectional	2021	Full−/part-time telework; social isolation	Labor judges	119	48.0 (8.4)	48.7	26.1	46.4 (8.9) h/week	16.1 (8.3)
Tareen 2024 (32)	India	Cross-sectional	2021	Full−/part-time WFH ≥ 1 h/day (computer-based); employed	Mixed	66	25.8 (6.9)	53.0	NR	NR	NR
Snodgrass 2022 (33)	International (46 countries): Australia (52.4%), United Kingdom (19.2%), United States (11.0%), other countries (17.7%)	Cross-sectional	2020–2021	Telework ≥75% of working time; computer/mobile use	Mixed	634	41.8 (11.2)	68.5	NR	NR	NR
Dockrell 2023 (38)	Ireland	Cross-sectional	2021	Computer-based telework; mandatory computer use	University staff	1,045	NR	67.7	NR	NR	NR
Sheeha 2025 (17)	Saudi Arabia	Cross-sectional	NR	WFH (high exposure); computer-based work	Office workers	336	39.8 (6.3)	100	27.1	4.4 (1.6) h/day (at work); 12.7 (7.0) h/week (at home)	9.2 (1.5)
Guler 2021 (37)	Turkey	Cross-sectional	2020	Full-time WFH; exclusion of hybrid workers	Office workers	194	34.0 (7.6)	49	24.9	9.5 (3.2) h/day	NR
Black 2022 (13)	Canada	Cross-sectional	2020–2021	Full-time WFH; ≥1 h continuous or >4 h/day computer use	Mixed	16	NR	75	NR	NR	NR
Narainsamy 2025 (39)	South Africa	Cross-sectional	2020	Full-time remote work (COVID-19); full-time employment	Academic staff	110	NR	59.1	NR	NR	NR
Florini 2023 (42)	Malta	Cross-sectional	Pandemic (12 months)	≥12 months telework	IT workers	459	NR	44.4	NR	NR	NR
Filho 2022 (20)	Brazil	Cross-sectional	2021	WFH during COVID-19	Judicial staff	934 pre-pandemic teleworkers: 258 pandemic-onset teleworkers: 676	45.9^f^	61.0	25.9^f^	39.8 h/week^f^	15.7^f^
Filho 2022 (14)	Brazil	Cross-sectional	2020	WFH due to social isolation; exclusion of pre-pandemic teleworkers	Labor justice workers	55	42.6 (7.5)	37	NR	38.1 (3.7) h/week	10.3 (6.9)
Filho 2024 (21)	Brazil	Cross-sectional	2021–2022	Partial telework (≤2 days/week in office); ≥1 year employment	Court employees	409 G1:153 G3:256	45.0^f^	63.1	25.8^f^	G1: 39.9 (5.4) h/week; G3: 39.5 (5.8) h/week	G1: 13.6 (7.3); G3: 15.3 (9.4)
Kamala 2024 (40)	India	Cross-sectional	2022–2023	WFH (IT sector); IT employment	IT workers	397	NR	45.6	NR	NR	NR
Janc 2023 (16)	Poland	Cross-sectional	2020–2021	WFH (<46%, 46–75, >75%); employee	University Staff	451 Teaching: 234 Administrative: 217	Teaching: 44.8 (10.3); Administrative: 42.5 (9.9)	81.8	NR	Computer use time (before vs. during COVID-19) Teaching: 8.2 (5.3) vs. 10.2 (3.2) h/day Administrative: 9.7 (4.1) vs. 10.2 (4.0) h/day	NR
Jabali 2026 (43)	Saudi Arabia	Cross-sectional	Pandemic	Remote work (COVID-19); ≥3 months; age ≥18	Mixed	978	NR	63.7	NR	NR	NR

NR; not reported. G1 – teleworking before the pandemic and at follow-up; G3 – teleworking initiated during the pandemic and continued at follow-up, WFH – work from home. ᵃ Male non-full teleworkers; ᵇ female non-full teleworkers; ᶜ male full/almost full teleworkers; ᵈ female full/almost full teleworkers. ^e^Data reported for 109 responders. ^f^Age, BMI, mean workload, and length of service data represent weighted means.

**Table 2:** Characteristics of the populations, exposures, and outcomes of the included studies.

**Study**	**MSD** **prevalence (%)**	**Most affected regions (%)**	**MSD Assessment Tool**	**Work-Related Assessment Tool**	**NOS**
Soto-Martínez 2025 (12)	82.3	Neck 62.7% Low back 56.8% Shoulders 45.0% Upper back 43.1%	Modified NMQ (musculoskeletal symptoms; 9 body regions; 10-month recall)	NASA-TLX (mental workload); ROSA (ergonomic risk, total score; sections A–C)	6
Ambrosio 2023 (31)	NR	NR	ODI (low back disability), VAS (pain intensity)	NR	8
Takasaki 2024 (36)	NR	NR	KDI (neck-related disability), RMDQ (low back disability), QuickDASH (upper extremity disability); LEFS (lower extremity function)	NR	7
Bilecik 2023 (41)	NR	NR	Before vs. pandemic NDI (neck disability), NRS (pain intensity)	NR	5
Houle 2021 (34)	NR	Neck pain 70.6%; Headache 61.5%	5-day follow-up NBQ (neck pain and disability), NRS (pain intensity)	NR	4
Rodríguez-Nogueira 2020 (35)	NR	Spine, upper and lower limbs	Before vs. during confinement Modified SNQ (musculoskeletal symptoms; 9 body regions; 12-month and 7-day recall)	NR	7
Filho 2023 (15)	NR	Neck, shoulders, low back	NMQ-Br + NRS (12 months vs. 7 days);	ROSA (ergonomic risk; total score and sections A–C) MUEQ (workstation, posture, job control, job demands, breaks, and social support)	6
Tareen 2024 (32)	NR	NR	Before vs. lockdown ODI (low back disability)	NR	3
Snodgrass 2022 (33)	91	NR	Modified NMQ (musculoskeletal symptoms) ODI (low back disability) VAS (pain intensity)	NR	4
Dockrell 2023 (38)	83	Neck 75.6% vs. 68.1%; Shoulders 69.7% vs. 60.4%; Low back 57.5% vs. 35.8%	Modified NMQ (musculoskeletal symptoms; 9 body regions; 3-month recall); laptop vs. desktop users	NR	5
Sheeha 2025 (17)	NR	NR	NR	ROSA (ergonomic risk; total score)	6
Guler 2021 (37)	NR	Neck, shoulders, upper back, low back	Before vs. pandemic Modified NMQ (musculoskeletal symptoms); VAS (pain intensity)	NR	5
Black 2022 (13)	NR	NR	NR	ROSA (ergonomic risk, total score; sections A–C)	3
Narainsamy 2025 (39)	81.8	Back 72% vs. 59.1% Neck 68% vs. 54.5% Shoulders 57% vs. 35.5%	Before vs. during pandemic Modified DMQ (musculoskeletal discomfort); SNQ (musculoskeletal symptoms) McCaffrey Pain Scale (pain intensity)	NR	5
Florini 2023 (42)	55.8	Back 39.7%; Neck/shoulders	Modified NMQ (musculoskeletal symptoms; 8 body regions; 12-month recall)	NR	6
Filho 2022 (20)	NR	Shoulders, neck, wrist/hand	Modified NMQ-Br (musculoskeletal symptoms; 12-month and 7-day recall); NRS (pain intensity)	MUEQ (Workstation, posture, control, demands, breaks, support)	7
Filho 2022 (14)	NR	6 month recall Shoulders 60%; Neck 54.5%; Upper back 40%; Low back 43.6%	Modified NMQ-Portuguese version (musculoskeletal symptoms; 6-month and 7-day recall)	ROSA (ergonomic risk, total score; sections A–D) MUEQ (Workstation, posture)	5
Filho 2024 (21)	NR	NR	Modified NMQ (musculoskeletal symptoms; 12-month and 7-day recall) NRS (pain intensity)	MUEQ Workstation, posture, control, demands, breaks, support	6
Kamala 2024 (40)	NR	Neck, shoulders, upper back, lower back	CMDQ (musculoskeletal discomfort questionnaire)	NR	6
Janc 2023 (16)	before vs. during COVID-19 Asymptomatic participants: teaching 17.9% vs. 16.7%; administrative 22.5% vs. 18.9%	NR	Modified NMQ (musculoskeletal symptoms)VAS (symptom severity)	ROSA (ergonomic risk, total score; sections A–D)	5
Jabali 2026 (43)	NR	Shoulders 37.0% Forearm 30.0% Mid-back 25.53% Low back 12.46%	Modified NMQ (musculoskeletal symptoms; 12 body regions; 11-month recall); pain intensity assessment	NR	8

*CMDQ – Cornell Musculoskeletal Discomfort Questionnaire; DMQ – Dutch Musculoskeletal Questionnaire; KDI – Katz Disability Index; LEFS – Lower Extremity Functional Scale; MUEQ – Maastricht Upper Extremity Questionnaire; NBQ – Neck Bournemouth Questionnaire; NDI – Neck Disability Index; NMQ – Nordic Musculoskeletal Questionnaire; NMQ-Br – Brazilian version of the Nordic Musculoskeletal Questionnaire; NMQ-Portuguese – Portuguese version of the Nordic Musculoskeletal Questionnaire; NRS – Numeric Rating Scale; NASA-TLX – National Aeronautics and Space Administration Task Load Index; ODI – Oswestry Disability Index; QuickDASH – Quick Disabilities of the Arm, Shoulder and Hand questionnaire; RMDQ – Roland–Morris Disability Questionnaire; ROSA – Rapid Office Strain Assessment; SNQ – Standardized Nordic Questionnaire; VAS – Visual Analogue Scale; NR – not reported*.

### 3.2 Population

Overall, the 21 studies included a total of 10,360 adults, with a predominance of female participants in most studies (range 37–100% female). The studies were conducted in Asia (17, 32, 36, 37, 40, 41, 43), Europe (16, 31, 35, 38, 42), North America (13, 34), South America (12, 14, 15, 20, 21), and Africa (39), with one study including an international sample (33). Age ranged from 25.8 (6.9) to 48.8 (8.7) years (12, 14–17, 20, 21, 31–33, 35–37, 41), with most participants aged 25–59 years (38–40, 42) and some studies including younger (18–24 years) (13, 40, 43) and older (≥60 years) individuals (38, 39, 43). BMI ranged from 20.6 to 27.1 kg/m^2^ (12, 15, 17, 20, 21, 34, 36, 37).

Most studies focused on a single occupational group (*n* = 16), while some studies included diverse occupational categories. Mixed occupational populations [*n* = 5; (13, 31–33, 43)], university and academic staff [*n* = 7; (12, 16, 29, 35, 38, 39, 41)], and office- and desk-based workers (*n* = 4) (17, 34, 36, 37) were the most frequently represented groups, followed by judicial employees (*n* = 4) (14, 15, 20, 21) and IT workers (*n* = 2) (40, 42).

### 3.3 Telework exposure and workload

Definitions of telework varied across studies. Most studies described telework as remote or home-based work (13, 17, 32–34, 38) performed using information and communication technologies (12, 14–16, 20, 21, 31, 35–37). Four studies quantified telework exposure: 100, >70%, 50–70%, 30–50%, or <30% home-based work (36), ≥75% telework (33), ≥1 h/day (32), and <46%, 46–75%, or >75% of working time (16). Four studies applied minimum telework duration criteria ranging from ≥1 week to ≥12 months as eligibility criteria rather than as a definition of telework (31, 34, 42, 43). Working hours were the most frequently reported measure and were expressed as mean weekly values in six studies (14, 15, 20, 21, 31, 41) and mean daily values in four studies (17, 34, 35, 37), which ranged from 12.7 (7.0) to 46.4 (8.9) hours per week and from 5.4 to 10.2 h per day. Other measures included the proportion of telework (33, 36) categorical workload ranges (32, 43) and sitting or computer use time (16, 35). Length of service was reported in six studies as a continuous variable, ranging from 5.3 to 17.0 years (12, 14, 15, 20, 21, 41). Two studies reported length of service using categorical measures (31, 40). Thirteen studies did not report this variable.

### 3.4 Assessment tools used across studies

Musculoskeletal symptoms were assessed using different instruments, most commonly Nordic-based questionnaires (NMQ/SNQ; n = 13). Other instruments were used to assess neck symptoms (NBQ, NDI), upper-limb symptoms (QuickDASH), low back symptoms (RMDQ, ODI), lower-limb symptoms (LEFS), and general musculoskeletal discomfort (CMDQ, KDI) (31–34, 36, 39–41).

Recall periods varied across studies using Nordic-based questionnaires. Twelve-month recall periods were most commonly reported (15, 20, 21, 35, 42), followed by 7-day recall periods (14, 15, 20, 21, 35). Less frequently, 6-month (14) and 10-month recall periods (12) were reported. Additional approaches included 4-week and 3-month recall periods and comparisons between pre-pandemic and pandemic periods (15, 16, 33, 37, 38). Musculoskeletal outcomes were reported as prevalence estimates (12, 14, 16, 20, 33, 34, 38, 39, 42, 43), symptom intensity scores (15, 16, 20, 21, 31, 33, 34, 37, 39, 41, 43), disability and functional outcomes (31–33, 36, 41), musculoskeletal discomfort (40), and the number of affected body regions (15, 20, 21, 36, 42). Among studies reporting overall MSD prevalence (12, 33, 38, 39, 42), values ranged from 55.8 to 91.0%. The neck was the most affected region, followed by the shoulders and the back (12, 15, 20, 37–43).

Ergonomic risk was assessed in 10 studies, most commonly using ROSA (*n* = 6) (12–17) and MUEQ (*n* = 4) (14, 15, 20, 21). All ROSA-based studies provided total scores. Four studies additionally included sections A–C (A: chair, B: monitor and telephone, and C: keyboard and mouse) (12–15), and two included combined B + C score (monitor, telephone, keyboard, and mouse) (13, 16). Regarding MUEQ, four studies included workstation, posture, control, demands, breaks, and support domains (14, 15, 20, 21). One study assessed workstation and posture domains only (14). Load Index (NASA-TLX) was used to evaluate perceived workload (12).

### 3.5 Quality assessment and risk of bias

The mean NOS score was 5.6 (range 3 to 8). The mean JBI score for cross-sectional studies was 6.4 (range, 3–8), with most studies classified as having a low risk of bias (10/19, 52.6%) The two prospective cohort studies had moderate (7/11) and high (4/11) risk of bias, respectively; two criteria were not applicable. The summarized quality assessment scores are presented in Table 2 and Supplementary Tables S1–S3.

### 3.6 Meta-analysis

#### 3.6.1 Musculoskeletal symptoms

Five studies comprising 2,215 participants contributed to the meta-analysis of musculoskeletal symptom prevalence. The proportion of women ranged from 48.7 to 67.7%, and participant age ranged from 25 to 64 years (12, 15, 35, 36, 40). The pooled prevalence was 54.9% (95% CI 20.8–85.0%) for neck symptoms, 52.8% (95% CI 34.1–70.7%) for shoulder symptoms, and 49.0% (95% CI 25.4–73.1%) for low back symptoms. Heterogeneity was high across all analyses (I^2^ = 98–99%) (Figure 2). The overall quality of evidence was rated as very low quality (ie, downgraded due to risk of bias, imprecision, and inconsistency) (Supplementary Table S4).

**Figure 2:**
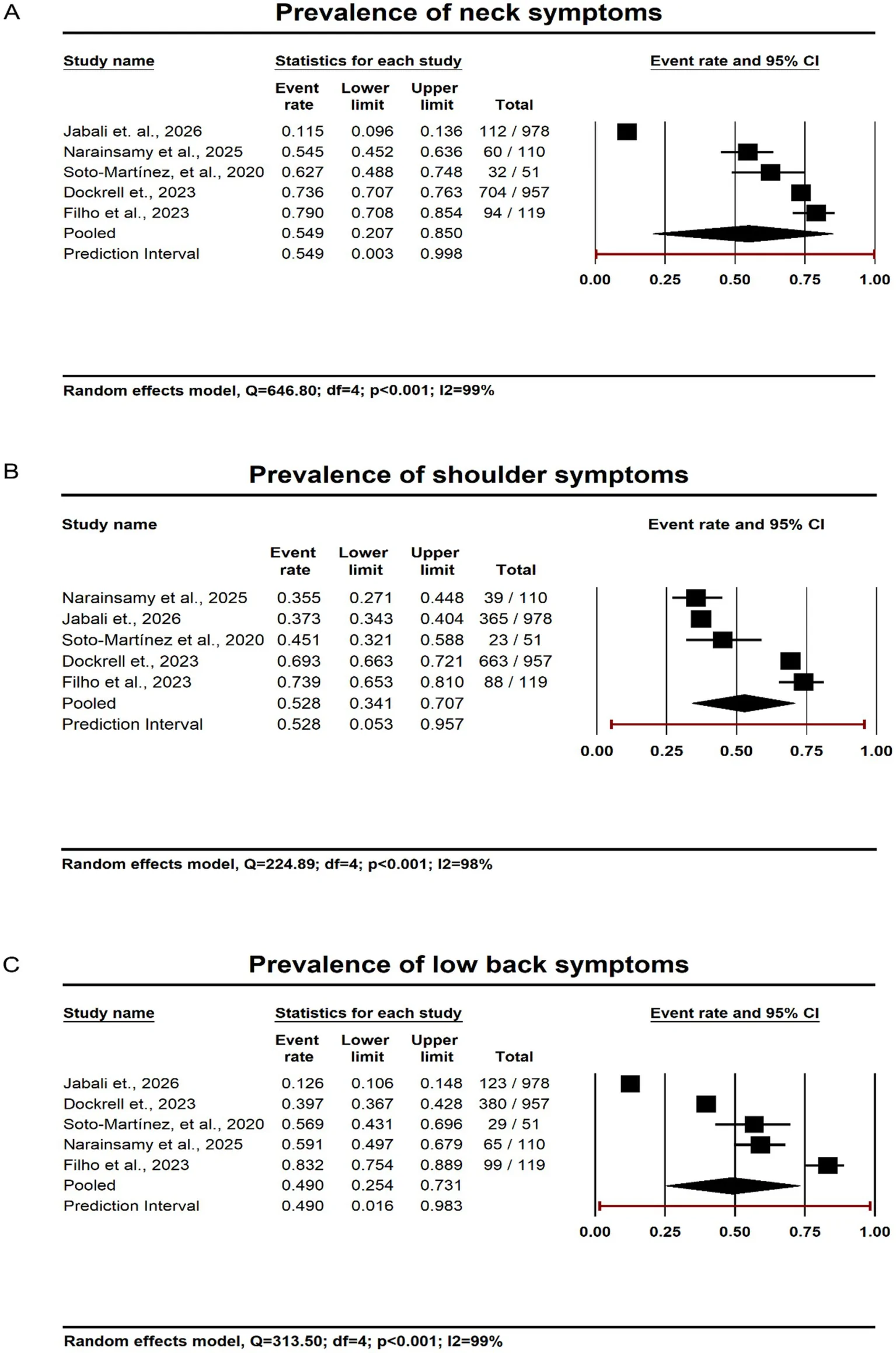
Forest plots of pooled prevalence estimates for neck **(A)**, shoulder **(B)**, and low back **(C)** symptoms among teleworkers.

#### 3.6.2 Ergonomic risk

Six studies (12–17) (Figure 3) comprising 1,086 participants contributed to the ergonomic risk analysis. Mean participant age ranged from 40 to 48 years. The pooled mean ROSA total score was 4.27 (95% CI 3.80–4.73; *p* < 0.001). Heterogeneity was high (*I*^2^ = 97.36%; Q = 189.18, df = 5, *p* = 0.001). Five studies comprising contributed to the analyses of ROSA sections A (*n* = 686), B (*n* = 692), and C (*n* = 692). The pooled mean score was 4.07 (95% CI 2.98–5.16; *p* < 0.001) for section A, 2.18 (95% CI 1.80–2.56; *p* < 0.001) for section B, and 3.04 (95% CI 2.69–3.39; *p* < 0.001) for section C. Heterogeneity was high across all analyses (section A: *I*^2^ = 98.71%, *Q* = 310.29, df = 4, *p* = 0.001; section B: *I*^2^ = 96.68%, *Q* = 120.38, df = 4, *p* = 0.001; section C: *I*^2^ = 91.91%, *Q* = 49.42, df = 4, *p* = 0.001) (Figures 3, 4). The certainty of evidence was low for the ROSA Total score and Sections B and C due to risk of bias and inconsistency, and very low for Section A due to risk of bias, inconsistency, and imprecision (Supplementary Table S5).

**Figure 3:**
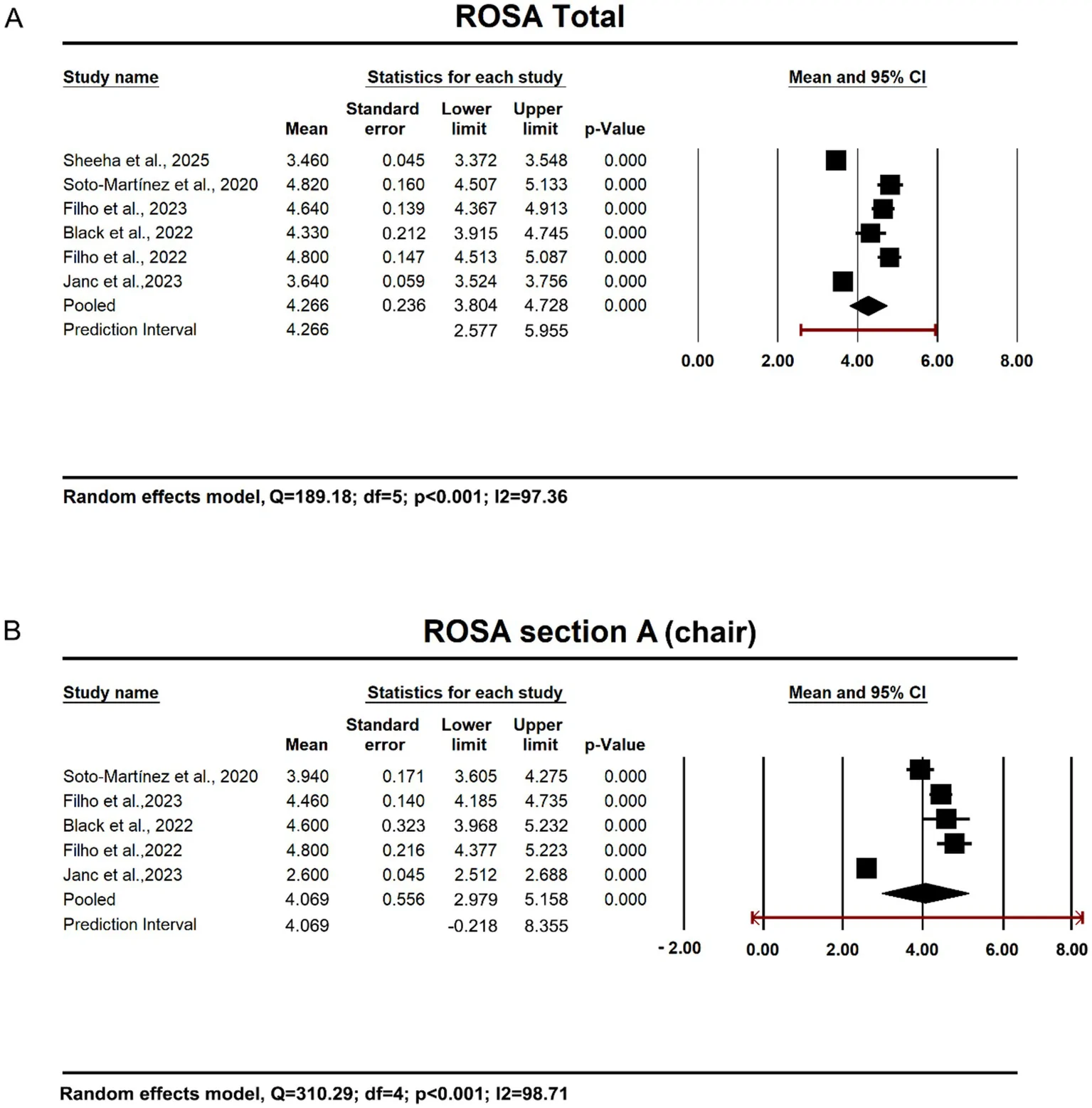
Forest plots of pooled mean ROSA Total **(A)** and ROSA Section A **(B)** scores among teleworkers.

**Figure 4:**
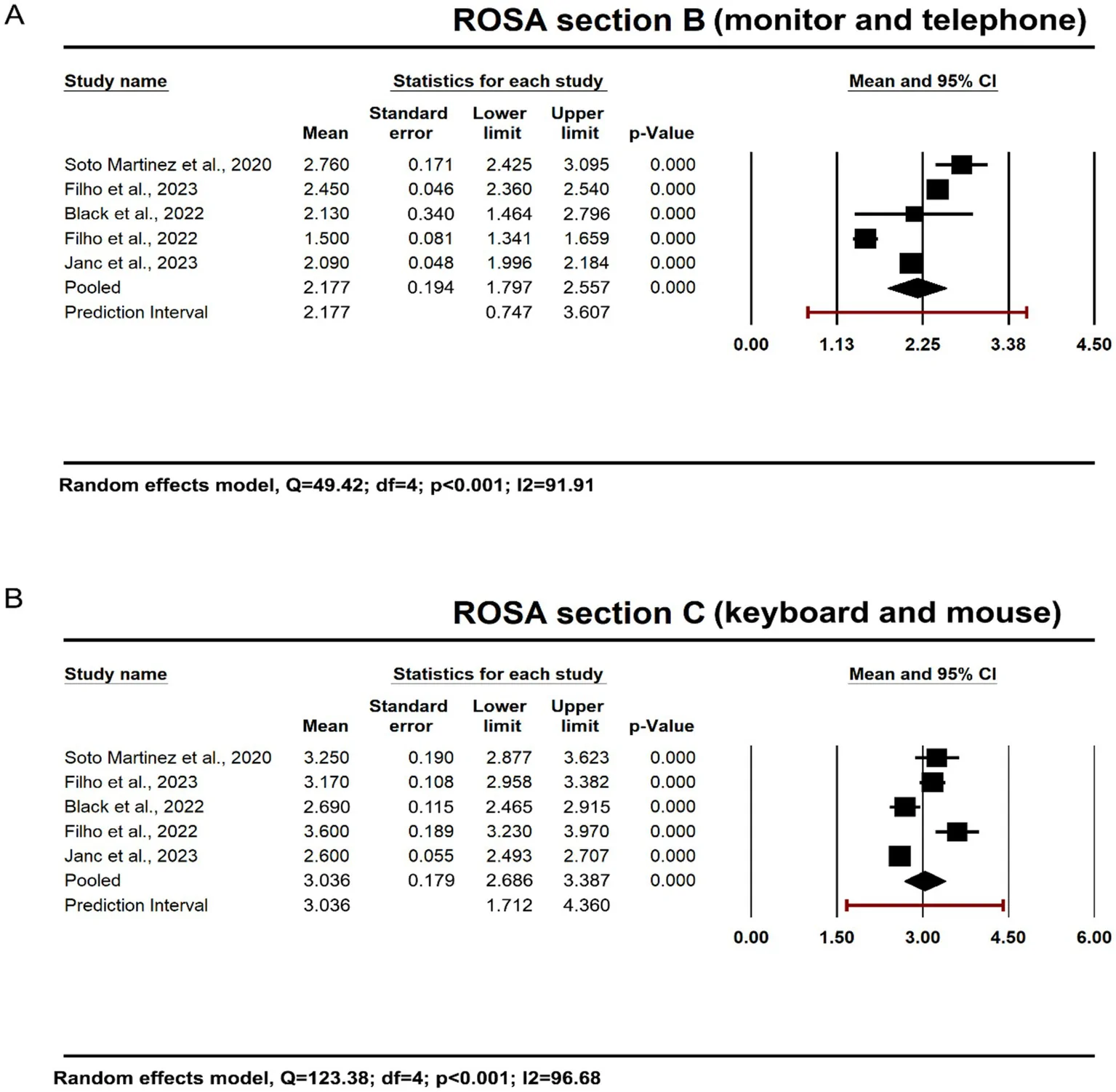
Forest plots of pooled mean ROSA section B **(A)** and ROSA Section C **(B)** scores among teleworkers.

### 3.7 Leave-one-out sensitivity analysis

Leave-one-out sensitivity analyses showed that the direction and magnitude of the pooled estimates remained largely unchanged after sequential removal of individual studies, indicating that the findings were not driven by any single study (Supplementary Figures S1–S3).

## 4 Discussion

This review aimed to quantify musculoskeletal symptom prevalence and ergonomic risk measured using validated assessment tools through a single-arm meta-analysis. Musculoskeletal symptoms were prevalent among teleworkers, with pooled prevalence estimates highest for the neck, followed by the shoulders and lower back. Across the six observational studies (including 1,086 participants), the pooled ROSA score indicated a moderate level of ergonomic risk among teleworkers (mean 4.27; PI: 2.58–5.95). Although this estimate did not reach the high-risk threshold of 5, the observed variability suggests that some teleworking populations may still exceed the intervention threshold, supporting the need for ongoing ergonomic surveillance and preventive strategies (44). According to GRADE, pooled evidence was of very low certainty for neck, shoulder, and low back symptom prevalence and low to very low certainty for ROSA-assessed ergonomic risk. Similar distributions of affected body regions have also been reported in office-based employees performing on-site work (18). A recent meta-analysis of office computer users in Sub-Saharan Africa reported lower prevalence estimates for neck (43.43%) and shoulder symptoms (33.71%) than those observed here, whereas the estimate for low back symptoms (46.48%) was comparable (3). Nevertheless, these estimates fall within the corresponding confidence intervals of our pooled prevalence estimates. Teleworkers and office-based workers share several risk factors, including prolonged sitting and extensive computer and ICT use (18). However, home-based workers may have less access to adequate workspace and resources to establish workstations that meet occupational safety and health standards, whereas office workplaces are expected to provide appropriate ergonomic conditions (45). Teleworkers are more likely to experience longer working hours, overtime, work during free time, irregular schedules, insufficient rest, and constant availability than office-based workers. Stress, mental strain, and sleep problems were slightly more common among teleworkers than office-based workers (46). According to the EWCS 2024, regular teleworkers (hybrid workers) had the highest risk of backache and upper-limb pain across working arrangements (47). The predominance of neck symptoms observed in our study is also consistent with Global Burden of Disease findings indicating a higher burden of neck pain among females than males (48). This observation is particularly relevant given that women represented the majority of participants in most included studies. Although Nordic-based questionnaires were the most frequently used instruments, considerable variability existed across studies in terms of recall periods, occupational populations (university staff, office workers, judicial employees, and IT workers), body regions assessed, and outcome reporting. Another potential source of between-study heterogeneity is the lack of a consistent definition of telework, with minimum exposure requirements ranging from 1 week to 12 months. Most evidence was based on self-reported measures, and several studies reported potential selection bias. These factors may partly account for the heterogeneity observed across prevalence estimates and may limit interpretation of the pooled findings. Home working conditions and access to ergonomic equipment may also have contributed to differences across studies. Several studies reported limited employer support for appropriate furniture and equipment, use of household furniture, and lack of a dedicated workstation (13, 32, 37, 38). In contrast, other populations had dedicated home office space and access to high-quality office furniture (14). Equipment provision may also depend on telework regulations, including requirements for workstation risk assessment and reimbursement of telework-related costs (45).

The chair section yielded the highest mean score among ROSA components, suggesting that telework does not eliminate chair-related ergonomic challenges associated with computer-based work. This finding is consistent with previous evidence identifying the chair as an important contributor to MSD risk in office-based workers (49). High ROSA-based ergonomic risk has also been reported in conventional office settings (50, 51), where up to 75% of office workers were classified as being at high risk (51). From an occupational perspective, ROSA may help identify specific workstation components that require ergonomic modification (46). Furthermore, Barros et al. (52) demonstrated that ROSA was responsive to ergonomic interventions, supporting its utility for both screening and monitoring ergonomic improvement (52).

Among the ergonomic risk tools, ROSA was most commonly used, followed by MUEQ. ROSA assessment methods varied across studies and included self-reporting (12, 16, 17), photographic analysis (13), and video observation (14, 15). This variability may have contributed to the substantial heterogeneity observed across ROSA estimates. This observation is supported by studies that applied both ROSA and MUEQ. While ROSA primarily assesses workstation-related ergonomic risk, MUEQ captures a broader range of work-related factors, including posture, job demands, breaks, and psychosocial aspects of work (14, 15, 21). Thus, the two instruments may be considered complementary, providing information on different dimensions of ergonomic risk. The wide prediction interval observed here, together with the broader scope of MUEQ, suggests that moderate ROSA-based ergonomic risk may coexist with a high prevalence of musculoskeletal symptoms among teleworkers. This finding may reflect the contribution of organizational and psychosocial factors beyond those captured by workstation-based assessments. However, this interpretation should be viewed with caution because evidence on MUEQ was derived from a small number of studies, largely from the same research group. These observations are also consistent with findings from the Fourth European Survey of Enterprises on New and Emerging Risks, which identified prolonged sitting (54%), repetitive movements (47%), increased work intensity (34%), and information overload (32%) as common occupational risk factors, particularly in office-based sectors (11). Several studies reported a direct association between screen time and symptom severity, consistent with evidence showing that longer working hours were associated with a modest increase in the risk of musculoskeletal pain (53). Greater telework exposure, including longer weekly working hours and after-hours work, was also associated with a higher prevalence of musculoskeletal symptoms. These observations are consistent with previous evidence linking telework with reduced physical activity and increased sedentary behavior (54).

The findings of this review have practical implications. Ergonomic assessment in teleworkers should extend beyond workstation-based screening, as musculoskeletal symptom burden appears to be influenced by physical, organizational, and psychosocial factors (18). Workstation-focused tools such as ROSA may be complemented by multidimensional instruments that capture organizational and psychosocial aspects of telework. Preventive strategies should prioritize the neck, lower back, and shoulders, the most consistently affected regions, and combine workstation adjustment, ergonomic education, regular breaks, and measures to reduce prolonged computer exposure and psychosocial strain. This multifactorial approach is also emphasized by Santos et al. (55). In a systematic review and meta-analysis of 24 randomized clinical trials, ergonomic interventions reduced musculoskeletal pain in the lower back, upper back, neck, wrist, and ankle. The authors highlighted the potential value of combining workstation modifications with cognitive strategies (e.g., mental workload management) and employee training.

To our knowledge, this is the first systematic review and meta-analysis to synthesize evidence on validated musculoskeletal assessment tools and ergonomic risk measures in teleworking populations. Additional strengths include the quantitative synthesis of both musculoskeletal symptom prevalence and ROSA outcomes across diverse teleworking populations. However, this review also has several limitations. First, the included evidence was dominated by cross-sectional studies, limiting causal inference. Therefore, the findings reflect prevalence and associations and do not establish a causal relationship between telework and musculoskeletal symptoms. Second, most studies relied on self-reported data, making the findings vulnerable to recall bias, subjective over- or under-reporting, and the absence of clinical verification of symptoms. Third, important methodological differences were observed in the assessment of musculoskeletal symptoms, particularly in relation to modified Nordic-based questionnaires, recall periods, and reporting formats. Furthermore, telework exposure was inconsistently defined across studies, ranging from general descriptions of remote work to quantitative thresholds and minimum duration criteria, which may have limited comparability between study populations. However, this variability also reflects the heterogeneous nature of contemporary telework, encompassing full-time, hybrid, and occasional work-from-home arrangements (47). Fourth, representativeness was limited in several studies because of sector-specific samples, low response rates, and possible volunteer bias. Fifth, important confounding factors, including psychological distress, general health status, and pandemic-related stressors, were not consistently controlled for. Sixth, the limited number of studies did not allow subgroup analyses. Publication bias could not be assessed because of the limited number of studies and cannot be ruled out. Finally, only a limited number of studies could be included in the meta-analysis, and evidence on MUEQ was derived from a small number of studies, largely from the same research group. Furthermore, substantial statistical heterogeneity was observed across pooled analyses, which may limit the precision and generalizability of the summary estimates. Most included studies were conducted during the COVID-19 pandemic, when telework was introduced rapidly and home workstations were often not adapted for prolonged computer use. Current telework is increasingly organized as hybrid work, with greater attention to work organization, equipment provision, and occupational health and safety (45, 46). These differences may require caution when applying the pooled estimates to current teleworking populations.

Although most studies were of moderate quality according to the Newcastle–Ottawa Scale and most cross-sectional studies were at low risk of bias according to JBI, GRADE certainty for the meta-analyzed outcomes was low or very low

## 5 Conclusion

Teleworkers frequently reported musculoskeletal symptoms, particularly in the neck, shoulders, and lower back, while pooled ROSA estimates indicated a moderate level of ergonomic risk. These findings suggest that workstation ergonomics alone may not fully explain musculoskeletal symptoms among teleworkers and that ROSA and MUEQ may provide complementary information on ergonomic and broader work-related factors. Substantial methodological variability across studies supports the need for more standardized and validated assessment approaches in teleworking populations.

## Data Availability

The original contributions presented in the study are included in the article/Supplementary material, further inquiries can be directed to the corresponding author/s.

## Footnotes

1 https://doi.org/10.17605/OSF.IO/EMVYG, accessed on 6 June 2026.
