# Supplementary material for: Musculoskeletal symptoms among teleworkers assessed using validated tools: a systematic review and single-arm meta-analysis
Source: Front Public Health. 2026 Sep 28;14:1941061. doi: 10.3389/fpubh.2026.1941061 (PMC13654979; doi:10.3389/fpubh.2026.1941061)
Supplement: Supplementary file 1 [file Supplementary_file_1.docx]

Supplementary Material

**Search strings**

*PubMed*

("Flexible Workplace"[Title/Abstract] OR "Hybrid Work"[Title/Abstract] OR "Remote Work"[Title/Abstract] OR "Telecommuting"[Title/Abstract] OR "Telework"[Title/Abstract] OR "non-teleworkers"[Title/Abstract]) AND ("musculoskeletal disorder"[Title/Abstract] OR "MSD"[Title/Abstract] OR "musculoskeletal health"[Title/Abstract] OR "musculoskeletal diseases"[MeSH Terms] OR "musculoskeletal injury"[Title/Abstract] OR "musculoskeletal pain"[Title/Abstract] OR "upper limb"[Title/Abstract] OR "upper extremity"[MeSH Terms] OR "neck pain"[Title/Abstract] OR "low back pain"[MeSH Terms] OR "back pain"[Title/Abstract] OR "shoulder pain"[Title/Abstract] OR "muscle pain"[Title/Abstract] OR "knee pain"[Title/Abstract] OR "foot pain"[Title/Abstract] OR "elbow pain"[Title/Abstract] OR "hip pain"[Title/Abstract] OR "hand pain"[Title/Abstract] OR "ergonomic risk"[Title/Abstract]) AND ("assessment tools"[Title/Abstract] OR "questionnaires"[Title/Abstract] OR "survey"[Title/Abstract] OR "ergonomic assessment"[Title/Abstract] OR "screening tools"[Title/Abstract] OR "observation methods"[Title/Abstract] OR "self-report instruments"[Title/Abstract] OR "ROSA"[Title/Abstract] OR "RULA"[Title/Abstract] OR "REBA"[Title/Abstract] OR "NMQ"[Title/Abstract] OR "CMDQ"[Title/Abstract] OR "Chronic Pain Grade"[Title/Abstract] OR "Brief Pain Inventory"[Title/Abstract] OR "MUEQ"[Title/Abstract] OR "Working Posture Analysing"[Title/Abstract] OR "Assessment of Repetitive Tasks"[Title/Abstract] OR "Revised Strain Index"[Title/Abstract] OR "OCRA"[Title/Abstract] OR "ULRA"[Title/Abstract]) NOT ("systematic review"[Publication Type] OR "review"[Publication Type] OR "meta-analysis"[Publication Type])

*Scopus*

TITLE-ABS-KEY("Flexible Workplace" OR "Hybrid Work" OR "Remote Work" OR "Telecommuting" OR "Telework" OR "non-teleworkers") AND TITLE-ABS-KEY("musculoskeletal disorder" OR "MSD" OR "musculoskeletal health" OR "musculoskeletal injury" OR "musculoskeletal pain" OR "upper limb" OR "neck pain" OR "low back pain" OR "back pain" OR "shoulder pain" OR "muscle pain" OR "knee pain" OR "foot pain" OR "elbow pain" OR "hip pain" OR "hand pain" OR "ergonomic risk") AND TITLE-ABS-KEY("assessment tools" OR "questionnaires" OR "survey" OR "ergonomic assessment" OR "screening tools" OR "observation methods" OR "self-report instruments" OR "ROSA" OR "RULA" OR "REBA" OR "NMQ" OR "CMDQ" OR "Chronic Pain Grade" OR "Brief Pain Inventory" OR "MUEQ" OR "Working Posture Analysing" OR "Assessment of Repetitive Tasks" OR "Revised Strain Index" OR "OCRA" OR "ULRA") AND DOCTYPE(ar)

Scopus popr

TITLE-ABS-KEY(("Flexible Workplace" OR "Hybrid Work" OR "Remote Work" OR "Telecommuting" OR "Telework" OR "non-teleworkers")

AND

("musculoskeletal disorder" OR "MSD" OR "musculoskeletal health" OR "musculoskeletal injury" OR "musculoskeletal pain" OR "upper limb" OR "neck pain" OR "low back pain" OR "back pain" OR "shoulder pain" OR "muscle pain" OR "knee pain" OR "foot pain" OR "elbow pain" OR "hip pain" OR "hand pain" OR "ergonomic risk")

AND

("assessment tools" OR "questionnaires" OR "survey" OR "ergonomic assessment" OR "screening tools" OR "observation methods" OR "self-report instruments" OR "ROSA" OR "RULA" OR "REBA" OR "NMQ" OR "CMDQ" OR "Chronic Pain Grade" OR "Brief Pain Inventory" OR "MUEQ" OR "OWAS" OR "Assessment of Repetitive Tasks" OR "Revised Strain Index" OR "OCRA" OR "ULRA"))

AND DOCTYPE(ar)

*Web of Science*

(TS=("Flexible Workplace" OR "Hybrid Work" OR "Remote Work" OR "Telecommuting" OR "Telework" OR "non-teleworkers")) AND (TS=("musculoskeletal disorder" OR "MSD" OR "musculoskeletal health" OR "musculoskeletal injury" OR "musculoskeletal pain" OR "upper limb" OR "neck pain" OR "low back pain" OR "back pain" OR "shoulder pain" OR "muscle pain" OR "knee pain" OR "foot pain" OR "elbow pain" OR "hip pain" OR "hand pain" OR "ergonomic risk")) AND (TS=("assessment tools" OR "questionnaires" OR "survey" OR "ergonomic assessment" OR "screening tools" OR "observation methods" OR "self-report instruments" OR "ROSA" OR "RULA" OR "REBA" OR "NMQ" OR "CMDQ" OR "Chronic Pain Grade" OR "Brief Pain Inventory" OR "MUEQ" OR "Working Posture Analysing" OR "Assessment of Repetitive Tasks" OR "Revised Strain Index" OR "OCRA" OR "ULRA")) AND (DT=(Article))

*EBSCO*

(TI ("flexible work" OR "flexible working" OR "hybrid work" OR "remote work" OR telecommuting OR telework)

OR

AB ("flexible work" OR "flexible working" OR "hybrid work" OR "remote work" OR telecommuting OR telework))

AND

(TI ("musculoskeletal disorder*" OR "musculoskeletal pain" OR "musculoskeletal injur*" OR "musculoskeletal health" OR "upper limb" OR "neck pain" OR "low back pain" OR "back pain" OR "shoulder pain" OR "muscle pain" OR "knee pain" OR "foot pain" OR "elbow pain" OR "hip pain" OR "hand pain" OR "ergonomic risk")

OR

AB ("musculoskeletal disorder*" OR "musculoskeletal pain" OR "musculoskeletal injur*" OR "musculoskeletal health" OR "upper limb" OR "neck pain" OR "low back pain" OR "back pain" OR "shoulder pain" OR "muscle pain" OR "knee pain" OR "foot pain" OR "elbow pain" OR "hip pain" OR "hand pain" OR "ergonomic risk"))

AND

(TI ("assessment tool*" OR questionnaire* OR survey OR "ergonomic assessment" OR "screening tool*" OR "observation method*" OR "self-report instrument*" OR ROSA OR RULA OR REBA OR NMQ OR CMDQ OR "Chronic Pain Grade" OR "Brief Pain Inventory" OR MUEQ OR OWAS OR "Assessment of Repetitive Tasks" OR "Revised Strain Index" OR OCRA OR ULRA)

OR

AB ("assessment tool*" OR questionnaire* OR survey OR "ergonomic assessment" OR "screening tool*" OR "observation method*" OR "self-report instrument*" OR ROSA OR RULA OR REBA OR NMQ OR CMDQ OR "Chronic Pain Grade" OR "Brief Pain Inventory" OR MUEQ OR OWAS OR "Assessment of Repetitive Tasks" OR "Revised Strain Index" OR OCRA OR ULRA))

**Supplementary Figure S1.** Leave-one-out sensitivity analysis for the pooled prevalence of musculoskeletal symptoms among teleworkers.

**Supplementary Figure S2.** Leave-one-out sensitivity analysis for ROSA total and ROSA section A scores among teleworkers.

**Supplementary Figure S3.** Leave-one-out sensitivity analysis for ROSA section B and ROSA section C scores among teleworkers.

**Supplementary Table S1.** Newcastle-Ottawa scale quality assessment

| Author  year | Design | S1 | S2 | S3 | S4 | C1A | C1B | O1 | O2 | O3 | Total |
| --- | --- | --- | --- | --- | --- | --- | --- | --- | --- | --- | --- |
| Soto-Martínez 2025 [12] | Cross-sectional | 0 | 0 | 1 | 0 | 1 | 1 | 1 | 1 | 1 | 6 |
| Ambrosio 2023 [32] | Cross-sectional | 0 | 1 | 1 | 1 | 1 | 1 | 1 | 1 | 1 | 8 |
| Takasaki 2024 [37] | Cross-sectional | 0 | 1 | 0 | 1 | 1 | 1 | 1 | 1 | 1 | 7 |
| Bilecik 2023 [42] | Prospective cohort | 0 | 1 | 1 | 0 | 0 | 0 | 1 | 1 | 1 | 5 |
| Houle 2021 [35] | Prospective cohort | 0 | 1 | 1 | 0 | 1 | 0 | 1 | 0 | 0 | 4 |
| Rodríguez-Nogueira 2020 [36] | Cross-sectional | 0 | 1 | 1 | 1 | 1 | 1 | 1 | 1 | 1 | 7 |
| Filho 2023 [15] | Cross-sectional | 0 | 0 | 1 | 1 | 1 | 0 | 1 | 1 | 1 | 6 |
| Tareen 2024 [33] | Cross-sectional | 0 | 0 | 0 | 1 | 0 | 0 | 1 | 0 | 1 | 3 |
| Snodgrass 2022 [34] | Cross-sectional | 0 | 0 | 0 | 1 | 0 | 0 | 1 | 1 | 1 | 4 |
| Dockrell 2023 [39] | Cross-sectional | 0 | 0 | 1 | 1 | 0 | 0 | 1 | 1 | 1 | 5 |
| Sheeha 2025 [17] | Cross-sectional | 0 | 1 | 0 | 1 | 1 | 0 | 1 | 1 | 1 | 6 |
| Guler 2021 [38] | Cross-sectional | 0 | 0 | 0 | 0 | 1 | 1 | 1 | 1 | 1 | 5 |
| Black 2022 [13] | Cross-sectional | 0 | 0 | 0 | 1 | 0 | 0 | 1 | 0 | 1 | 3 |
| Narainsamy 2025 [40] | Cross-sectional | 0 | 1 | 0 | 1 | 0 | 0 | 1 | 1 | 1 | 5 |
| Florini 2023 [43] | Cross-sectional | 0 | 0 | 0 | 1 | 1 | 0 | 1 | 1 | 1 | 6 |
| Filho 2022 [20] | Cross-sectional | 0 | 0 | 1 | 1 | 1 | 1 | 1 | 1 | 1 | 7 |
| Filho 2022 [14] | Cross-sectional | 0 | 0 | 1 | 1 | 0 | 0 | 1 | 1 | 1 | 5 |
| Filho 2024 [21] | Cross-sectional | 0 | 0 | 1 | 1 | 1 | 0 | 1 | 1 | 1 | 6 |
| Kamala 2024 [41] | Cross-sectional | 0 | 0 | 0 | 1 | 1 | 1 | 1 | 1 | 1 | 6 |
| Janc 2023 [16] | Cross-sectional | 1 | 0 | 0 | 1 | 0 | 0 | 1 | 1 | 1 | 5 |
| Jabali 2026 [44] | Cross-sectional | 0 | 1 | 1 | 1 | 1 | 1 | 1 | 1 | 1 | 8 |

Abbreviation

S1	Representativeness of the sample

S2	Sample size

S3	Non-respondents

S4	Ascertainment of exposure

C1A	Control for the most important factor

C1B	Control for additional factors

O1	Assessment of outcome

O2	Outcome assessment / follow-up*

O3	Statistical test

Total	Total NOS score

**Supplementary Table S2**. Methodological Quality of the Included Prospective Cohort Studies Assessed Using the JBI Critical Appraisal Checklist

| Author  Year | Design | Q1 | Q2 | Q3 | Q4 | Q5 | Q6 | Q7 | Q8 | Q9 | Q10 | Q11 | Total |
| --- | --- | --- | --- | --- | --- | --- | --- | --- | --- | --- | --- | --- | --- |
| Bilecik 2023 [42] | Prospective cohort | N/A | N/A | U | Y | N | N | Y | Y | N | N | Y | 4 |
| Houle 2021 [35] | Prospective cohort | N/A | NA | Y | Y | Y | N | Y | Y | N | Y | Y | 7 |

**Q1.** Were the two groups similar and recruited from the same population?
**Q2.** Were the exposures measured similarly to assign people to both exposed and unexposed groups?
**Q3.** Was the exposure measured in a valid and reliable way?
**Q4.** Were confounding factors identified?
**Q5.** Were strategies to deal with confounding factors stated?
**Q6.** Were the groups/participants free of the outcome at the start of the study (or at the moment of exposure)?
**Q7.** Were the outcomes measured in a valid and reliable way?
**Q8.** Was the follow-up time reported and sufficient to be long enough for outcomes to occur?
**Q9.** Was follow-up complete, and if not, were the reasons for loss to follow-up described and explored?
**Q10.** Were strategies to address incomplete follow-up utilized?
**Q11.** Was appropriate statistical analysis used?

**Supplementary Table S3**. Methodological Quality of the Included Cross-Sectional Studies Assessed Using the JBI Critical Appraisal Checklist

| Author  year | Design | Q1 | Q2 | Q3 | Q4 | Q5 | Q6 | Q7 | Q8 | Total |
| --- | --- | --- | --- | --- | --- | --- | --- | --- | --- | --- |
| Soto-Martínez 2025 [12] | Cross-sectional | Y | Y | U | Y | Y | Y | Y | Y | 7 |
| Ambrosio 2023 [32] | Cross-sectional | Y | Y | Y | Y | Y | Y | Y | Y | 8 |
| Takasaki 2024 [37] | Cross-sectional | Y | Y | Y | Y | Y | Y | Y | Y | 8 |
| Rodríguez-Nogueira 2020 [36] | Cross-sectional | Y | Y | Y | U | Y | Y | U | Y | 6 |
| Filho 2023 [15] | Cross-sectional | Y | Y | Y | Y | Y | N | Y | Y | 7 |
| Tareen 2024 [33] | Cross-sectional | Y | Y | Y | U | Y | N | Y | Y | 6 |
| Snodgrass 2022 [34] | Cross-sectional | y | y | y | U | y | y | Y | Y | 7 |
| Dockrell 2023 [39] | Cross-sectional | Y | Y | Y | U | Y | Y | U | Y | 6 |
| Sheeha 2025 [17] | Cross-sectional | Y | Y | U | Y | Y | Y | Y | Y | 7 |
| Guler 2021 [38] | Cross-sectional | Y | Y | Y | U | Y | Y | Y | Y | 7 |
| Black 2022 [13] | Cross-sectional | U | Y | Y | U | N | N | U | Y | 3 |
| Narainsamy 2025 [40] | Cross-sectional | Y | Y | Y | Y | Y | N | Y | Y | 7 |
| Florini 2023 [43] | Cross-sectional | Y | Y | U | Y | Y | Y | Y | Y | 7 |
| Filho 2022 [20] | Cross-sectional | Y | Y | Y | Y | Y | Y | Y | Y | 8 |
| Filho 2022 [14] | Cross-sectional | Y | Y | Y | U | Y | N | U | Y | 5 |
| Filho 2024 [21] | Cross-sectional | Y | Y | Y | Y | Y | N | Y | Y | 7 |
| Kamala 2024 [41] | Cross-sectional | U | Y | U | Y | Y | N | Y | Y | 5 |
| Janc 2023 [16] | Cross-sectional | Y | Y | Y | U | Y | N | Y | Y | 6 |
| Jabali 2026 [44] | Cross-sectional | U | Y | U | Y | Y | Y | Y | Y | 5 |

Q1. Were the criteria for inclusion in the sample clearly defined?
Q2. Were the study subjects and the setting described in detail?
Q3. Was the exposure measured in a valid and reliable way?
Q4. Were objective, standard criteria used for measurement of the condition?
Q5. Were confounding factors identified?
Q6. Were strategies to deal with confounding factors stated?
Q7. Were the outcomes measured in a valid and reliable way?
Q8. Was appropriate statistical analysis used?

**Supplementary Table S4**. GRADE Evidence Profile With Summary of Findings for Musculoskeletal Symptom Prevalence

| **Outcome** | **Studies,**  **n** | **Population** | **ROB** | **Inconsistency** | **Indirectness** | **Imprecision** | **Publication Bias** | **Overall Certainty of Evidence** |
| --- | --- | --- | --- | --- | --- | --- | --- | --- |
| Prevalence of neck symptoms | 5 | 2215 | Serious^a^ | Serious^b^ | Not serious^c^ | Serious^d^ | Not assessed^e^ | Very Low |
| Prevalence of shoulder symptoms | 5 | 2215 | Serious^a^ | Serious^b^ | Not serious^c^ | Serious^d^ | Not assessed^e^ | Very Low |
| Prevalence of low back symptoms | 5 | 2215 | Serious^a^ | Serious^b^ | Not serious^c^ | Serious^d^ | Not assessed^e^ | Very low |

GRADE, Grading of Recommendations Assessment, Development and Evaluation; ROB, risk of bias.

**^a^**Serious risk of bias due to limitations in sample representativeness and non-response.

**^b^**Serious inconsistency due to considerable heterogeneity (I² = 98%–99%), variation in prevalence estimates, and wide prediction intervals.

**^c^**No serious indirectness; populations and outcomes were applicable to the review question.

**^d^**Serious imprecision due to wide 95% CIs and substantial uncertainty in prevalence estimates.

**^e^**Publication bias was not assessed because fewer than 10 studies were included.

**Supplementary Table S5.** GRADE Evidence Profile With Summary of Findings for Ergonomic Risk

| Outcome | Studies,  n | Population | ROB | Inconsistency | Indirectness | Imprecision | Publication Bias | Overall Certainty of Evidence |
| --- | --- | --- | --- | --- | --- | --- | --- | --- |
| ROSA Total | 6 | 1086 | Serious^a^ | Serious^b^ | Not serious^c^ | Not serious^d^ | Not assessed^e^ | Low |
| ROSA Section A | 5 | 686 | Serious^a^ | Serious^b^ | Not serious^c^ | Serious^d^ | Not assessed^e^ | Very Low |
| ROSA Section B | 5 | 692 | Serious^a^ | Serious^b^ | Not serious^c^ | Not serious^d^ | Not assessed^e^ | Low |
| ROSA Section C | 5 | 692 | Serious^a^ | Serious^b^ | Not serious^c^ | Not serious^d^ | Not assessed^e^ | Low |

^a^Serious risk of bias due to limitations in representativeness and non-response.

**^b^**Serious inconsistency (I² = 91.9%–98.7%) with wide prediction intervals.

**^c^**No serious indirectness; populations and outcomes were applicable to the review question.

**^d^**Serious imprecision for ROSA Section A due to the wide 95% CI.

**^e^**Publication bias was not assessed because fewer than 10 studies were included.
